# Treatment trends for undescended testis and impact of guideline changes a medical health care analysis of orchidopexy and cryptorchidism in Germany between 2006 und 2020

**DOI:** 10.1007/s00345-024-05095-x

**Published:** 2024-06-25

**Authors:** Marcus Sondermann, Viktoria Menzel, Angelika Borkowetz, Martin Baunacke, Johannes Huber, Nicole Eisenmenger, Christian Thomas, Katharina Boehm

**Affiliations:** 1 Department of Urology, Universitätsklinikum Carl-Gustav-Carus Dresden, Technische Universität Dresden, Dresden, Germany; 2 Department of Urology, Universitätsklinikum Gießen-Marburg, Philipps Universität Marburg, Marburg, Germany; 3 Reimbursement Institute, Hürth, Germany

**Keywords:** Orchidopexy, Cryptorchidism, Non-descensus testis, Undescended testis, Epidemiology, Real-life data, Health service

## Abstract

**Background:**

The last decades revealed new scientific knowledge regarding the fertility and potential malignancy of undescended testis AQ2(UDT). Accordingly, many guidelines changed their recommendation concerning timing of therapy, with the goal of an earlier time of surgery.

**Methods:**

We analyzed the number of new diagnosis and performed surgeries in predefined age groups provided by the obligatory annual reports of German hospitals in the reimbursement.INFO”-tool between 2006 and 2020.

**Results:**

Overall, 124,741 cases were analyzed. We showed a slight increase in performed surgeries in the first year by 2% per year with a main increase till 2011, a constant number of surgeries between first and 4th year and a decrease of surgeries between 5 and 14th year of living with a main decrease till 2009 by 3% per year.

**Conclusion:**

Even if our results illustrate an increasing adaption of the guideline’s recommendation, there is still a significant number of patients who receive later treatment. More research about the reasons and circumstances for the latter is needed.

**Supplementary Information:**

The online version contains supplementary material available at 10.1007/s00345-024-05095-x.

## Introduction

Undescended testis (UDT) describes a malposition of one or both testes, specifically a non-scrotal position. The prevalence in normal weight born males is between 1.8 up to 8.4% and may increase up to 48% in males with low birth weight or prematurity status [1–3]. A physiological descensus is possible in 7% of patients within the first twelve months of living [4, 5], although the likelihood decreases after sixth months [4].

An untreated UDT is related with subfertility and an increased risk for testicular malignancy [6–8]. The risk for infertility is higher in patients with bilateral UDT. The goal of orchidopexy is to decrease the risk for malignancy, move the testicles in position for self-examination, help to increase testicular volume and maybe fertility [2, 9–12]. An untreated UDT is associated with a 2.2-time higher risk for testicular cancer [9, 13].

Accordingly, the German, American, and European guidelines changed their recommendation regarding timing of treatment in the second decade of the twenty-first century.

With this in mind, the goal of our study was to analyze the effect of German guideline changes in 2016 on the rate of orchidopexy, stratified according to age in a large cohort of patients.

We hypothesized that the number of orchidopexy in early age is increasing and the number of patients treated at older age is decreasing, both in relation to the specific birth rate.

## Methods and analysis

In Germany, hospitals are legally obligated to report data concerning diagnosis and treatment of their patient in an annual report since 2005 [14]. The OPS and ICD-data are transferred to the Institute of Hospital Remuneration and subsequently to the German Federal Statistical Office (Destatis). We extracted the OPS (German adaption of the International Classification of Procedures in Medicine) orchidopexy with funiculysis (OPS 5-624.4) by the “reimbursement.INFO”-tool (RI Innovation GmbH, Hürth, Germany) [15]. This tool includes all diagnoses encoded by ICD and all cases of surgery by OPS performed in German hospitals either by age or hospital. The data are restricted to inpatient cases.

Hereby we were able to extract the number of performed surgeries by year and by specific age groups (younger than 1, between 1 and 4, between 5 and 9, between 10 and 14, 15 – 19, 20 – 25, 25 – 29, above the age of 30 years). Age groups were predefined in the “reimbursement.INFO”-tool and could not be adapted.

Similarly, we collected the data by ICD (International Statistical Classification of Diseases and Related Health Problems) for nondescensus testis (ICD: Q53). To evaluate the incidence of orchidopexy for UDT, birth rate data were ascertained using the nationwide Destatis database [16]. The incidence was computed by number of orchidopexies per number of boys born. Additionally, the incidence was calculated for the specific age groups. The primary data are publicly accessible, anonymized and processed by the latest declaration of Helsinki. Therefore, no ethical statement was needed.

For our analyses, three age groups were evaluated: (1) younger than 1 year, (2) 1 to 4 years and (3) 5 to 14 years. The analyses were performed for the overall patient group, as well for the age specific subgroups. Descriptive analyses were performed and an augmented Dick-Fuller-Test (ADF) for changes over time was used [17]. The augmented Dick-Fuller-Test states that a time series is stationary if the null hypothesis can be rejected. This can be interpretated as no change over time. In case of a change over time (according to the ADF-test), a difference-sign test was used to test for increase or decrease and a linear model was fitted for the level of change [18]. Due to the reduction of random errors the ADF-Test performs better in this data set than the Dick-Fuller-Test (DF). In addition, we performed a Joinpoint regression to test for changes over time [19, 20]. In a Joinpoint regression, also known as change point regression, a dataset is divided into subsets with a unique linear trend inside the subset. This way a point of change in a non-stationary time period can be detected and quantified by a slope and an intercept [20]. This results in specific time points when a trend is changing. Other statistical methods cannot detect a concrete point of a change in a regression.

All data we collected in Excel and analysis were performed using R (Version 2023.03.0 Build 386, packages: dplyr, tidyr, ggplot2, gtsummary, ljr, randtest) [21–24].

## Results

### Analyses of surgical rate in the overall cohort

The birth rate of male newborn remained stable between 2006 and 2021, with a median (md) of 359,337 [interquartile range (IQR); 346,882; 400,096] births per year. A temporary rise of male newborn rates by 15% can be seen between 2013 and 2015.

**Fig. 1:**
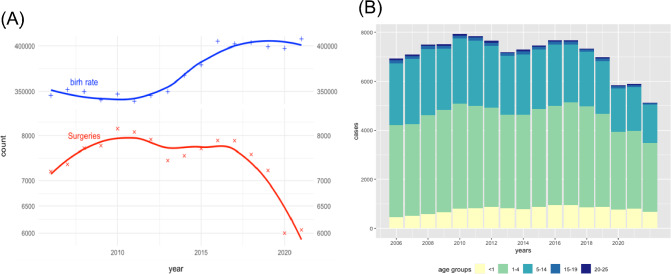
Number of surgeries over Time. The number of surgeries (red line) per year and the male births (blue line) per year are pointed out in (**a**). Here the ordinate is in logarithmic scaling. We can see a gentle rise of male birth between 2013 and 2015. The number of orchidopexies stayed stable over time between 2006 and 2019 and dropped since 2020. (**b**) Total numbers of surgeries for all age groups. Here an almost constant number of surgeries over time can be seen

Similarly, the total number of orchidopexies per year remained stable until 2019 (md: 7,633 [7,316, 7,880]). This results in a median incidence of 2.1% [1.9, 2.2] of all newborn males per year who undergo orchidopexy. After 2019 the number of surgeries dropped (md: 6,000 [5,648; 6,029,5]) by 12%. The yearly incidences of performed surgeries were depicted in Fig. 1(**a**).

In total 124,741 cases could be analyzed between 2006 and 2022 (Details in Supplementary Table 1). Of all patients 12,306 (10%) were younger than 13 months, 62,416 (52%) were 1–4 years old, 27,959 (23%) were 5 to 9 years old and 10,382 (9%) were 10 to 14 years old. Patients older than 14 years (*n* = 3543; 3%) were excluded from further analyses.

### Age specific subgroup analyses

**Fig. 2:**
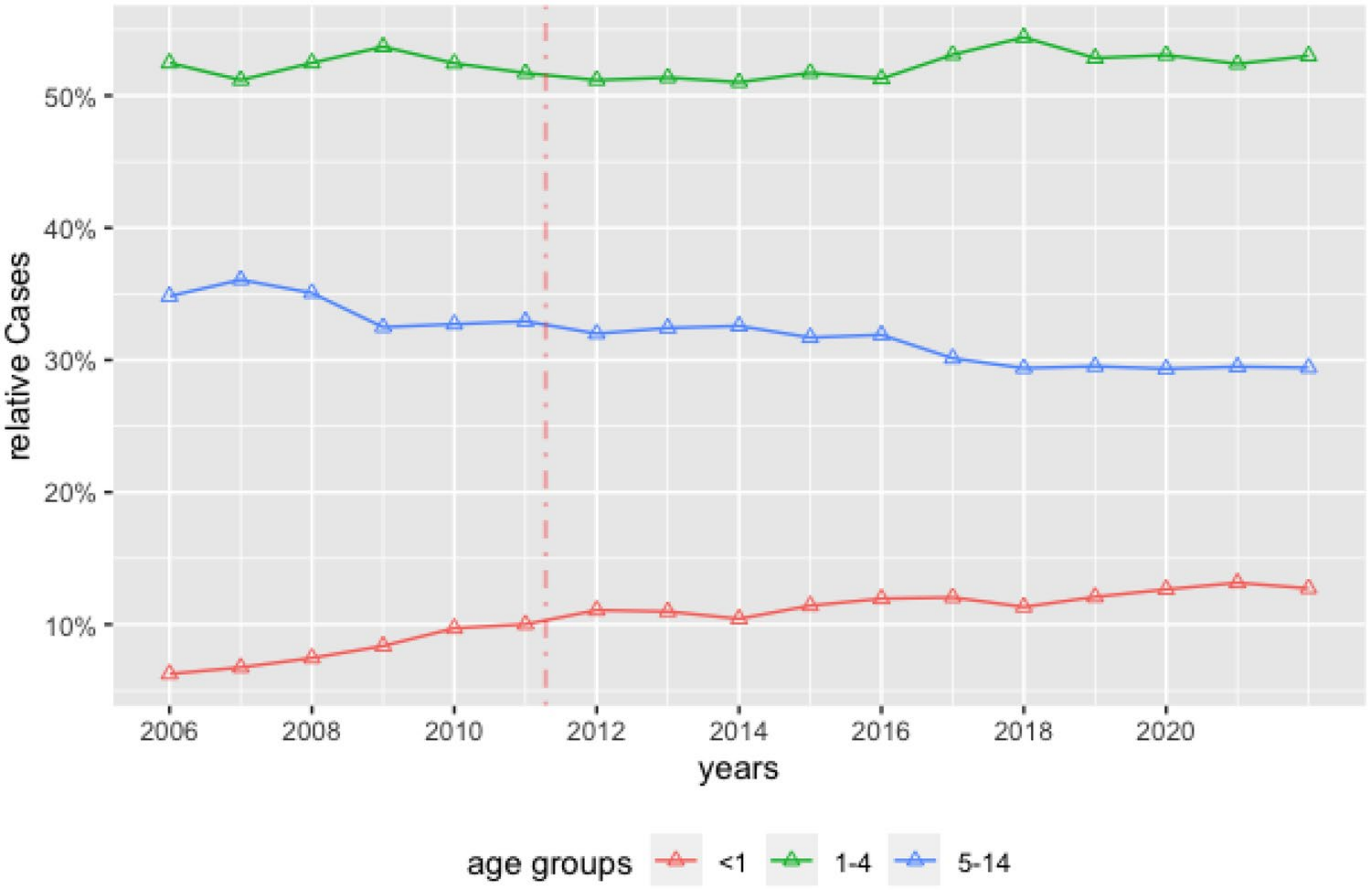
Joinpoint-Regression. Number of orchidopexies over time between 2006 and 2022 stratified by age group. In (**a**) the relative development in the essential groups under one (red), between one and four (green) and between five and fourteen years (blue) of age at surgery are depicted. Here the slight increase in surgeries under one year of age can be seen. The vertical dotted red line represents the change of growth by Joinpoint regression

12,980 children in our cohort were younger than one year at surgery. (10.4% [IQR 662–872]). The percentage of surgeries performed before the first birthday showed a nonstationary behavior (ADF-Test: p = 0.86) with an increase of cases (Trend-Test p < 0.01) by 2% per year (R^2^ = 0.89, p-value < 0.001). The Joinpoint regression analysis revealed a turning point in 2011, showing an increase in surgical rate by 11% per year and a slowing increase after 2011. The rate of surgery increased from 6.2% to 13% in this age group. This is pictured by the red dotted line in Fig. 2.

In the second age group, 52% ([IQR 51; 53]; n = 65,223) of children were between one and four years of age. For this subgroup, no trend in the incidence of surgery could be shown (ADF-Test: p = 0.9; Difference trend Test: p = 0.19). In addition, a Joinpoint regression was not adding further information. This is depicted in Fig. 2 (green line).

Finally, 39,899 [IQR: 3767–4143] patients were between 5 and 14 years old. The incidence of orchidopexy in this age group was decreasing over time from 34 to 29% (ADF-Test: p = 0.11; Difference Trend Test: p = 0.002) [30; 33]. The Joinpoint regression analyses shows the main decrease prior 2009 by 3% per year and a constant behavior after 2009. This is depicted in Fig. 2 (blue line).

Overall, two-third of cases were performed prior the fourth year of age.

## Discussion

Our results support our hypothesis, that age at surgery decreases for patients with UDT. This may be due to new data on benefits of early surgery of maldescended testis.

First, our data show a stable number of orchidopexy rates as well as overall cases over time.

Furthermore, we found an increase in surgeries within the first year of life with a main increase from 2006 to 2011. Moreover, percentage of surgeries performed in older age (older than 5 years), are statistically significantly decreasing. In general, the group with the highest risk for long term side effect is shrinking. Finally, number of surgeries performed at the age from one to 5 remained stable.

Several studies have shown that the therapy for UDT should be completed between the 12th and 18th month of living [6, 25]. This is based on the beforementioned risks for testicular cancer, fertility, and the unlikelihood of spontaneous descensus testis after an age of six months. Accordingly, the German, American, and European guidelines changed their recommendation regarding timing of treatment in the second decade of the twenty-first century. The German guideline states that the therapy of cryptorchidism should be complected within the first year of life. According to the AUA (American urology association) guideline the therapy should be finalized until the age of 18 months [26]. Real life data show that only 40% of patients with undescended testis in the US completed the therapy within this timespan [27]. The same study showed slightly better guideline accordance in urban areas. In rural territories the median age at therapy was 59 months with a rate of therapy under the age of 18 months of 29%. In 70% of cases the therapy is delayed at least 6 months [28]. In 2008 only 4% of children finalized the therapy at their first birthday in Germany [10, 29]. These rates are comparable for other western industrialized countries [30].

Our results show that only one of 6 orchidopexies (16%) in Germany is performed before the first birthday and hereby matches the guideline recommendation. This seems to be a nonacceptable rate of delayed surgeries, although routine examination should prevent this effect. In Germany regular examinations at specific time points in childhood are required by law. However countries like Italy, Sweden or New Zeeland manage to have lower rates of delayed surgery with the same system of routine examination after birth [30–32].

It may be that a high number of surgeries is performed slightly after the first birthday. This might be associated with a statistically decreased risk of anesthesia after the first birthday and might tempt surgeons and anesthesiologists to schedule surgery slightly later than recommended [33, 34]. Due to the origin of data, we could not further analyze the age structure of the very heterogenous group aged one to four years. Another explanation is a lack of awareness for the correct timing of therapy of UDT in healthcare professionals and/or parents. In a study by Boehme et al. only two-third of health care professionals could state the correct timing of treatment. Although the knowledge was higher amongst urologists (79%), pediatrics (81%), and pediatric surgeons (89%) [10]. This issue can be addressed with a higher awareness and alertness in the preventive examinations in childhood or a better interdisciplinary cooperation between surgeons and pediatricians. Overall, the acceptance of the guideline recommendation seems to be high [35].

However, a major part of surgeries is performed prior to fourth birthday and almost all surgeries performed before the ninth's birthday. This leaves a minor rate of patients undergoing surgery after the 10th birth year, who finally are at even higher risk for testicular malignancy.

Another explanation for the relatively high number of patients receiving delayed surgery might be that a high proportion of those children present with secondary ascension. A congenital undescended testis (cUDT) must be differentiated from secondary ascension. The incidence of secondary ascension after surgery is low [36]. The incidence of secondary acquired undescended testis (saUDT) is described between 13 and 44% [37, 38]. The proportion of saUDT increases by age. Almost half of orchidopexies after the 12th month of life were performed due to a saUDT [39]. Furthermore, this indication to orchidopexy dominated after the age of three [38]. Unfortunately, it was not possible to distinguish between patients with primary UDT and secondary ascension in our analyses.

We could show a drop of surgeries beginning in 2020 with no rebound effect for the following two years. It is possible, that pediatric examinations were neglected during COVID pandemic. Data on pediatric examinations during COVID are rare and mostly based on small cohorts. But a decrease of elective surgeries up to 75% was measurable [40]. A decrease of cases is also overserved in emergency visits with increased adverse events in pyelonephritis [41] and decreased rates of outpatient visits in cancer screening [42–44].

A change in the guidelines might have a positive impact on the knowledge about standard of care. Changing the guideline affects the standard of care, as reported for circumcisions [45]. But it remains unclear whether the guideline change, or the primary data of the scientific community leads to the difference. Our data support the latter. Scientific data may have affected the change in standard of care in first line, as the main increase of orchidopexies within the first year of life was found till 2011. The change in guidelines around 2016 seems to absolve the standard of care and amplify the conclusions from scientific data. Regarding our data we must also keep in mind a possible effect of a bow wave. This means all children had earlier surgery and fewer patients were left for later therapy. This leads to an increase in cases for a couple of years.

Our study helps to understand that a non-negligible number of patients may not receive surgery at an early timepoint. Healthcare professionals should start to plan surgical treatment at the age of 6–9 months. In times of reduced surgical capacity this may help to ensure timely treatment.

Additionally, it may be helpful to differentiate between primary UDT and secondary UDT to make sure that the German system of regular examinations, that are required by law, is helping to improve healthcare in a significant way. That is also one of our limitations. We could not distinguish the etiology of UDT. A difference between aUDT, saUDT and cUDT was not possible. It is possible that a substantial part of the “late” surgeries were secondary ascensions. The documentation of primary or secondary UDT could be addressed by quality reports that are required anyway and help for further research.

Secondly, the hospital data saved in the “reimbursement.INFO”-tool are only retrievable in predefined age structures. So, we were not able to analyze the data in a more detailed age structure. A cut off at month 18 might reaffirm our analysis and bring up more arguments.

Lastly and most relevant, an increasing number of surgeries were performed as outpatient procedures. As the hospitals are obliged to report only their inpatient data, we had no access to outpatient procedures, although a non-negligible number of surgeries is performed as outpatient procedures in children. In 2008 around 4.500 orchidopexies were performed as outpatient procedures in Germany [46]. In our observation 7.569 surgeries were performed as inpatient procedures in Germany. This leads to a two third rate of surgeries in an inpatient setting; although other studies observed a 50% ratio [47]. Outpatient surgeries in children are limited to a cooperative social structure as well as healthy children with few comorbidities [44]. UDT is a disease with higher rates of comorbidity. This might explain a higher rate of delayed surgeries. However, Lippert et al. found a similar rate of children younger than a year (12% 0–13 months at surgery vs 10.4% in our cohort) [46]. Additionally, the rate of children older than 3 years was lower in our cohort of inpatient children (60% > 3 years vs 32% ≥ 4 years). These results suggest that children in an outpatient setting may not undergo surgery at an earlier point of time compared to those in an inpatient setting.

In summary, we can provide a current report of timing in orchidopexies in a very large cohort. The benefits of early orchidopexy in children are well known among the involved physicians and the data show a good trend. But more education, awareness and campaigns are mandatory. The goal of pediatrics, urologists and pediatric surgeons should be to increase the number of surgeries before the first birthday and to make effort to improve data about secondary ascension and its clinical impact.

## Supplementary Information

Below is the link to the electronic supplementary material.

Supplementary file1 (DOCX 18 KB)

## Data Availability

The datasets used and analyzed during the current study are available from the corresponding author on reasonable request.
