# Supplementary material for: Treatment trends for undescended testis and impact of guideline changes a medical health care analysis of orchidopexy and cryptorchidism in Germany between 2006 und 2020
Source: World J Urol. 2024 Jun 25;42(1):386. doi: 10.1007/s00345-024-05095-x (PMC11199275; doi:10.1007/s00345-024-05095-x)
Supplement: Supplementary file 1 — Supplementary file1 (DOCX 18 KB) [file 345_2024_5095_MOESM1_ESM.docx]

# Supplementary

|  | OPS: 5-624.4 | | | | ICD: Q53 | | | |
| --- | --- | --- | --- | --- | --- | --- | --- | --- |
| year | **overall** | **age <1** | **age 1-4** | **age 5-14** | **overall** | **age < 1** | **age 1-4** | **age 5-14** |
| 2006 | 7193 | 450 | 3774 | 2505 | 7379 | 456 | 4032 | 2891 |
| 2007 | 7349 | 496 | 3761 | 2651 | 7541 | 516 | 4005 | 3020 |
| 2008 | 7707 | 575 | 4044 | 2703 | 7939 | 670 | 4244 | 3025 |
| 2009 | 7764 | 650 | 4169 | 2522 | 7979 | 753 | 4432 | 2794 |
| 2010 | 8165 | 793 | 4282 | 2671 | 8242 | 889 | 4421 | 2932 |
| 2011 | 8084 | 809 | 4180 | 2662 | 8158 | 946 | 4359 | 2853 |
| 2012 | 7907 | 876 | 4046 | 2530 | 7777 | 941 | 4148 | 2688 |
| 2013 | 7433 | 816 | 3818 | 2410 | 7392 | 876 | 3950 | 2566 |
| 2014 | 7539 | 786 | 3846 | 2456 | 7461 | 861 | 3992 | 2608 |
| 2015 | 7697 | 879 | 3981 | 2440 | 7740 | 997 | 4084 | 2659 |
| 2016 | 7883 | 942 | 4042 | 2515 | 8017 | 1076 | 4166 | 2775 |
| 2017 | 7879 | 949 | 4183 | 2374 | 7707 | 1009 | 4181 | 2517 |
| 2018 | 7569 | 857 | 4118 | 2224 | 7462 | 972 | 4100 | 2390 |
| 2019 | 7217 | 872 | 3813 | 2131 | 6926 | 971 | 3752 | 2203 |
| 2020 | 6000 | 759 | 3184 | 1760 | 5924 | 816 | 3256 | 1852 |
| 2021 | 6059 | 797 | 3175 | 1787 | 5969 | 927 | 3192 | 1850 |
| 2022 | 5296 | 674 | 2807 | 1558 | 5381 | 782 | 2974 | 1625 |
| Table 1 Characteristics of Patients by OPS and ICD | | | | | | | | |
